# Early activation of CD4+ and CD8+ T lymphocytes by myelin basic protein in subjects with MS

**DOI:** 10.1186/s12967-015-0715-6

**Published:** 2015-11-02

**Authors:** Borros Arneth

**Affiliations:** Institute of Laboratory Medicine and Pathobiochemistry, Molecular Diagnostics, University Hospital of the Universities of Giessen and Marburg UKGM, Justus Liebig University Giessen, Feulgenstr. 12, 35392 Giessen, Germany

## Abstract

**Background:**

Multiple sclerosis is the most common autoimmune disorder affecting the central nervous system. In this study, whole blood samples were analyzed for activation capacity and the activatability of CD4+ and CD8+ T-lymphocytes by human total myelin basic protein (MBP), human MBP 104–118 fragment, and guinea pig MBP 68–82 fragment.

**Methods:**

Whole blood samples from healthy human subjects were compared with samples from patients with multiple sclerosis (MS). In particular, the expression of CD69, a surface marker of T-lymphocyte activity, was measured via flow cytometry before and after 14 h of incubation with human total MBP, MBP 104–118 fragment and/or guinea pig MBP 68–82 fragment. The results were compared between 15 patients with MS and 15 healthy subjects.

**Results:**

In response to all three MBP forms, CD4+ and CD8+ T-lymphocytes from patients with MS demonstrated greater activatability than those from healthy subjects. These results indicate that in patients with MS, latent pre-activation to MBP epitopes results in an increased activation capacity of T-lymphocytes.

**Conclusion:**

This effect may occur because immunization against MBP (at least in a subset of patients) plays a pathophysiological role in MS pathogenesis. Alternatively, this result may represent a non-specific, bystander autoimmune phenomenon.

## Background

The symptoms of multiple sclerosis (MS) are considered to represent signs of an autoimmune disease [1]. In MS, the autoimmune response is directed against the envelope and/or surface components of nerve sheaths (i.e., myelin sheaths, oligodendrocytes) [1–3].

MS is a human disease that is closely related to the animal model of experimental autoimmune encephalitis (EAE) [4]. In this animal model, mice are immunized by an injection of myelin basic protein (MBP) and subsequently develop an MS-like relapsing disease pattern.

However, whether immunization against MBP also occurs in humans as part of MS development is so far unknown [2, 3]. Indeed, immunization against human MBP is associated with the occurrence of pre-activated T-lymphocytes specific to MBP, and several authors have described immune reactions and autoimmune diseases as consequences of T-lymphocytes remaining from former immunizations [4–7].

To investigate this topic in greater detail, whole blood samples from patients with MS and healthy control subjects were incubated with three different MBP types (human total MBP, human MBP 104–118 fragment, and guinea pig MBP 68–82 fragment) overnight. As a positive control, whole blood samples were preincubated with concanavalin A, and as a negative control, whole blood samples from the same subjects were preincubated with human albumin. Then, cells incubated with or without human MBP were examined by means of flow cytometry, and the proportions of activated CD4+ and CD8+ T-lymphocytes from healthy control volunteers and patients with MS were evaluated.

## Methods

### Patients

As part of routine blood tests, 15 patients with MS and 15 healthy subjects underwent two withdrawals of EDTA whole blood in the morning (2 times, 7.5 ml each draw). Characteristics of the patients are given in Table 1. All patients provided informed consent. The study was approved by the relevant review board.

**Table 1:** Patient and healthy control subject characteristics

Demographic variables	MS patients	Healthy controls
Gender (men/women)	6/9	6/9
Age (years)	36.5 (6.0)	32 (5.5)
Years of education	13.0 (3.1)	13.0 (2.2)
Neurological assessment		
Disease duration (months)	32.2 (9.6)	–
Time since last relaps (months)	5.8 (6.5)	–
EDSS	0.92 (0.85)	–
Type of MS		
Relapsing remitting	13	–
Primary progressive	2	–
Secondary progressive	–	–

### Lymphocyte activation

For each individual, the whole blood sample was typically divided into six 1 ml aliquots/tubes. Concanavalin A (Sigma-Aldrich, Bremen, Germany) was added to tube 1 (positive control). Tube 2 was left untreated. Tube 3 was treated with human albumin as a negative control. Human total MBP, human MBP 104–118 fragment and guinea pig MBP (68–82 fragment, all Sigma-Aldrich, Bremen, Germany) were added to tubes 4, 5 and 6, respectively. All proteins were added to a final concentration of 2 µg/ml. All experiments (incubations and flow cytometric analysis) were performed in duplicate for each subject.

### Incubation period

Incubation of the whole blood samples was performed with (tubes 4, 5, and 6) and without (tubes 2) the addition of MBP followed by flow cytometric analysis. All whole blood samples were incubated at 37 °C (98.6 °F) for 14 h. Approximately once every 2 h, the samples were gently mixed to keep the whole blood in motion.

### Staining of cells for flow cytometry

A flow cytometric assay (Fastimmune) from Becton–Dickinson (BD Bioscience, Heidelberg, Germany) was used. The following extracellular epitopes and/or markers were evaluated by flow cytometry: CD3, CD4, CD8, and CD69. Staining was performed after incubation according to the manufacturer’s instructions (FAST immune assay, Becton–Dickinson, San Diego, CA, USA). An antibody mixture (Becton–Dickinson, San Diego, CA, USA) was added to 50 µl of human whole blood. The antibody mixture contained antibodies against CD4, CD8, CD3, and CD69. CD3 is expressed predominantly by T-lymphocytes, CD69 is an early activation marker, CD4 is a marker for T-helper cells (CD4+ T-lymphocytes), and CD8 is a marker for cytotoxic T-lymphocytes (CD8+ T-lymphocytes). The antibody specific for CD69 was labeled with R-phycoerythrin (PE), as PE is the most sensitive fluorochrome available for staining. The CD8−, CD3−, and CD4− specific antibodies were labeled with fluorescein isothiocyanate (FITC), peridinin chlorophyll protein (PerCP), and allophycocyanin (APC), respectively. The blood samples were incubated with the antibody mixture for 20 min at room temperature.

### Erythrocyte lysis

After a 20 min incubation period at room temperature with the antibody mixture, erythrocytes were lysed with 450 µl of BD lysis buffer (1×) according to the manufacturer’s instructions (Becton–Dickinson). After 20 min in lysis buffer, the samples were analyzed by flow cytometry.

### Flow cytometry

The samples were analyzed using a FACSCalibur (Becton–Dickinson, San Diego, CA) flow cytometer. The fluorescence values of FITC, PerCP, APC, and PE were measured and graphically overlaid. The cells were initially gated on the basis of the CD3 PerCP signal and side scatter. The CD3-positive population was considered to be the T-lymphocyte population. A total of 30,000 events in the T-lymphocyte gate were recorded per sample.

Gating was performed based on side scatter and CD3 expression for T-lymphocytes. Within this lymphocyte gate, CD4+ and CD8+ T-lymphocytes were separated on the x-axis. On the y-axis, the CD69 marker was plotted versus the CD4 and CD8 markers to represent the T-lymphocyte activation markers on each of the diagrams. The proportions of activated CD4+ and CD8+ T-lymphocytes were determined as the percentage of double-positive CD4+/C69+ T-lymphocytes and/or as the proportion of double-positive CD8+/CD69+ T-lymphocytes.

### Measurement evaluation

To evaluate the acquired data, the CD69 signal was compared separately to the CD4 and CD8 signals. The CD69 intensity was plotted against the CD4 and CD8 signals. The results were further analyzed with a four-field division, which yielded double-positive cells (upper right, UR), double-negative cells (lower left, LL), and cells in which only one of the markers was expressed (lower right, LR and upper left, UL). Therefore, the percentage of activated T-lymphocytes in each subpopulation (CD4+ or CD8+) was determined. The percentage of activated cells corresponded to the quotient of double-positive cells (UR) divided by the sum of the single-positive cells and the double-positive lymphocytes for each population (UR + LR). Thus, the quotients q4 and q8 were calculated for both CD4+ and CD8+ T-lymphocytes as follows q = UR/(UR + LR).

### Statistical analysis and normalization for baseline activation

The untreated blood samples consistently displayed a few weakly activated T-lymphocytes. To account for these cells in each experiment, the difference between the quotients of the treated and untreated blood samples from each subject were calculated. These differences were termed Δq4 = q4 − q40 and Δq8 = q8 − q80 for the CD4+ and CD8+ lymphocytes, respectively. Thus, the values obtained for q4 and q8 represented the percentages of activated T-lymphocytes in a treated sample (q4, q8) after the subtraction of background activation (q40, q80).

To investigate the influence of MBP in a mathematical/statistical way, the proportions of activated CD4+ and/or CD8+ T-lymphocytes from samples incubated with and without MBP were compared using Student’s t test and the Wilcoxon test.

In addition, the mathematical differences in activated T-lymphocytes were calculated for incubation with and without the specific peptide added (Δq4, Δq8).

## Results

**Fig. 1:**
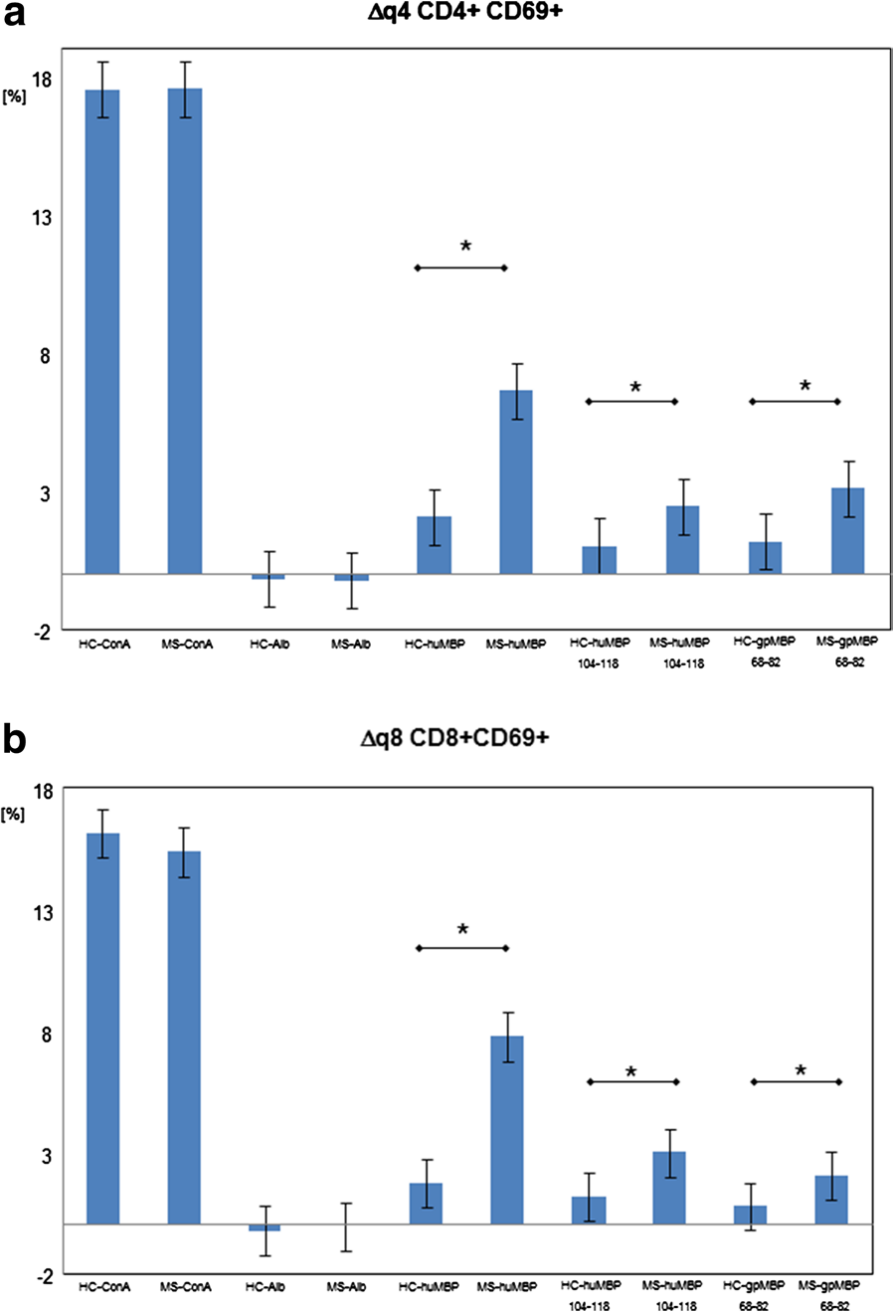
Activated cell populations of CD4+ T-cells Δq4 (**a**) and/or of activated CD8+ T-cells Δq8 (**b**) between the MBP-treated and the respective untreated samples. Significantly more activated T-lymphocytes were observed in samples from MS patients following an overnight incubation with MBP compared to samples from healthy controls (HCs). The effect was stronger for human total MBP than for human MBP 104–118 fragment an than fot guinea pig MBP 68–82 fragment. HC-ConA/HC-Alb/HC-huMBP/HC-huMBP114/HC-gpMBP68–82: Blood from HC incubated with concanavalin A, human albumin, human total MBP, huMBP104–118 peptide and/or guinea pig MBP 68–82 peptide. MS-ConA/MS-Alb/MS-huMBP/MS-huMBP114/MS-gpMBP68–82: blood from MS patients incubated with concanavalin A, human albumin, human MBP, human MBP104–118 peptide and/or guinea pig MBP 68–82 peptide (*significantly different, p < 0.01)

Figure 1 illustrates the differences between Δq4 (Fig. 1a) and Δq8 (Fig. 1b).

Figure 1a shows the differences in CD4+ T-lymphocyte activation between patients with MS (MS multiple sclerosis) and healthy control subjects (HC healthy controls); the numbers of activated CD4+ T-lymphocytes in samples incubated with and without the different MBPs (Δq4) are shown. Figure 1b shows the differences in Δq8 (CD8+) T-lymphocyte activation between patients with MS and healthy subjects (analogous to Fig. 1a, * statistical significant increase p < 0.01).

The samples from healthy subjects demonstrated up to 2 % more activated T-lymphocytes following the different MBP overnight incubations. However, the samples from patients with MS clearly demonstrated many more activated T-lymphocytes; these patient samples showed up to 10 % (mean 7 %) more activated CD4+ and CD8+ T-lymphocytes following overnight MBP incubation. There was no significant difference between the numbers of activated CD4+ and/or CD8+ T-lymphocytes.

Duplicate samples from the same subject always showed a high degree of consensus (always <2 % difference).

**Fig. 2:**
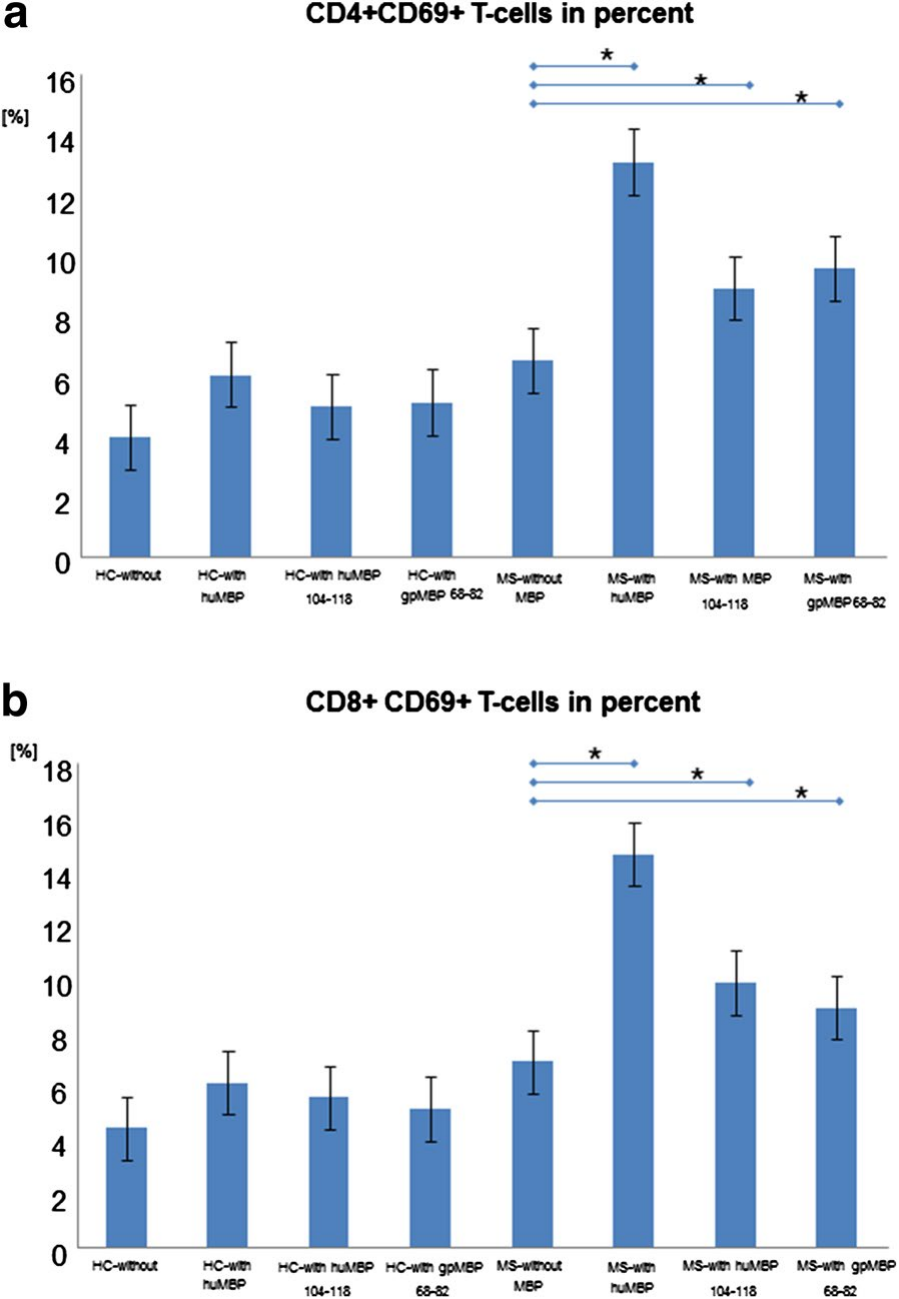
The percentages of activated CD4+ T-lymphocytes (**a**) and/or CD8+ T-lymphocytes (**b**) among treated and untreated cell aliquots. A significant increase in the number of activated T-lymphocytes was observed in the whole blood from patients with MS compared to in whole blood from healthy subjects following an overnight incubation with MBP. In comparison, samples from healthy subjects exhibited significantly fewer activated T-lymphocytes following incubation with the different MBPs. (*significantly different, p < 0.01). HC-without: blood from healthy controls (HC) without proteins/peptides added; MS-without: blood from MS patients without proteins/peptides added. HC-with total huMBP, huMBP104–1184, gpMBP68–82: blood from HC incubated with human MBP, human MBP104–118 peptide and/or guinea pig MBP 68–82. MS-with total huMBP, huMBP104–118, gpMBP68–82: blood from MS patients incubated with human MBP, human MBP104–118 peptide and/or guinea pig MBP 68–82

Figure 2a and b present the percent changes in the number of activated T-lymphocytes following overnight incubation with and without the different MBPs for CD4+ (Fig. 2a) and CD8+ (Fig. 2b) T-lymphocytes. H-without indicates healthy subjects and incubation without MBP. H-with indicates healthy subjects and incubation with MBPs (human total MBP, MBP 104–118 fragment, and/or guinea pig MBP 68–82 fragment). MS-without indicates MS-patients and incubation without any MBP. Finally, MS-with indicates MS-patients and incubation with the different MBPs (human total MBP, MBP 104–118 fragment, and/or guinea pig MBP 68–82 fragment).

In samples from healthy subjects, there were small increases in the numbers of both activated CD4+ lymphocytes and activated CD8+ T-lymphocytes. Compared to this small to moderate increase, there was a much stronger increase in the numbers of activated CD4+ and CD8+ T-lymphocytes following overnight MBP incubation in whole blood samples from patients with MS. For all three tested MBPs, this increase in activated CD4+ and CD8+ T-lymphocytes was statistically significant (p < 0.01). However, this increase in activated T-cells was most prominent following incubation with human total MBP, followed by human 104–118 fragment; the smallest increase was observed following incubation with guinea pig MBP 68–82 fragment (human total MBP > huMBP-104–118 > gpMBP-68–82).

## Discussion

Prior to MBP stimulation, blood samples from patients with MS exhibited only a slightly higher amount (2 %) of CD69+ T-lymphocytes compared with those from healthy subjects. This effect was not detectable in samples from all patients.

In contrast, the percentage of CD69+ T-lymphocytes was increased following MBP stimulation in samples from almost all patients with MS. The effect was stronger if total human MBP was used compared to either human MBP 104–118 fragment or guinea pig MBP 68–82 fragment.

In blood samples from healthy individuals, the effect of MBP stimulation on the proportion of activated T-lymphocytes was lower and was not detectable in the samples from a majority of subjects.

The results presented here demonstrate that T-lymphocytes from patients with MS respond very differently to MBP compared with T-lymphocytes from healthy subjects.

While the T-lymphocytes from healthy subjects demonstrated a relatively small response to MBP (or even no response), the reaction was significantly stronger in T-lymphocytes from patients with MS.

If we assume that the T-lymphocyte response is significantly stronger following the second contact with the same antigen or epitope (see Stryer, Janeway, and Kuby) [1, 8, 9], the results of this study suggest that previous contact with MBP may contribute to the development of MS in vivo.

Our results suggest that, at least in a subset of patients with MS, there is latent pre-activation of CD4+ and CD8+ T-lymphocytes towards MBP epitopes. Moreover, these findings are in agreement with the current opinion regarding MS pathogenesis [10–15].

## Conclusion

Pre-activation of CD4+ and CD8+ T-lymphocytes to MBP seems to be present in several MS patients. Furthermore, our results demonstrate that MBP is a major auto-antigen in patients with MS.

However, it remains unclear whether this result is a pathogenically relevant step in MS development or whether it is a non-specific secondary reaction.

The autoimmune response to MBP as an auto-antigen may constitute a pathogenetically significant immune response against an endogenous auto-antigen [2, 3]. In addition, this phenomenon may also be non-specific and driven via a bystander autoimmune response.

However, the evidence points toward the former hypothesis and is strengthened by the similarities between MS and the EAE animal model. In this model, MBP immunization is a required step for EAE development, and EAE is currently the best animal model of human MS disease [4].

It is also possible that MBP stimulates a non-specific, bystander autoimmune response, as pre-activation of T-lymphocytes against MBP was not detectable in all patients with MS. Thus, this relevant question cannot be fully answered in this context.

## References

[1] Janeway C, Travers P, Walport M, Shlomchik M (2001). Immunobiology: the immune system in health and disease.

[2] Allegretra M, Nicklas J, Sriram S, Albertini R (1990). T-cells responsive to myelin basic protein in patients with multiple sclerosis. Science.

[3] Meinl E, Weber F, Drexler K, Morelle C, Ott M, Saruhan-Direskeneli G, Goebels N, Ertl B, Jechart G, Giegerich G (1993). Myelin basic protein-specific T lymphocyte repertoire in multiple sclerosis. Complexity of the response and dominance of nested epitopes due to recruitment of multiple T cell clones. J Clin Invest.

[4] Constantinescu C, Faroogi N, O’Brien K, Gran B (2011). Experimental autoimmune encephalomyelitis (EAE) as a model for multiple sclerosis (MS). Br J Pharmacol.

[5] Waksman BH (1988). Autoimmunity in demyelinating diseases. Ann N Y Acad Sci.

[6] Zinkernagel RM, Bachmann MF, Kuendig TM, Oehen S, Pircher HP, Hengartner H (1996). On immunological memory. Annu Rev Immunol.

[7] Zinkernagel RM (2003). On natural and artificial vaccinations. Annu Rev Immunol.

[8] Stryer L. Biochemistry. 7th ed. New York: 2012.

[9] Goldsby RA, Kindt TJ, Osborne BA (2000). Kuby Immunology.

[10] Kaufmann SHE (1993). Immunity to intracellular bacteria. Annu Rev Immunol.

[11] Katz DH, Benacerraf B (1972). The regulatory influence of activated T cells on B cell responses to antigen. Adv Immunol.

[12] McFarlin, McFarland (1982). Multiple sclerosis (first of two parts). Engl J Med.

[13] Zamvil S, Mitchell D, Moore A, Kitamura K (1986). Steinman L and Rothbard J T-cell epitope of the autoantigen myelin basic protein that induces encephalomyelitis. Nature.

[14] Hafiler D, Benjamin D, Burks J, Weiner H (1987). Myelin basic protein and proteolipid protein reactivity of brain- and cerebrospinal fluid-derived T cell clones in multiple sclerosis and postinfectious encephalomyelitis. J Immunol..

[15] Fallis RJ, Powers ML, Sy MS, Weiner HL (1987). Adoptive transfer of murine chronic relapsing autoimmune encephalomyelitis. Analysis of basic protein-reactive cells in lymphoid organs and nervous system of donor and recipient animals. J Neuroimmunol.

